# Critical mapping of epidemiology training in graduate programs in collective health in Brazil: challenges and perspectives

**DOI:** 10.1590/1980-549720250004.supl.1

**Published:** 2025-11-17

**Authors:** Ana Paula Muraro, Amanda de Moura Souza, Cassia Maria Buchalla, Enirtes Caetano Prates Melo, Gerusa Gibson, João André Tavares Álvares da Silva, Lívia Teixeira de Souza Maia, Maria Aparecida Araújo Figueiredo, Sérgio Viana Peixoto, Taynãna César Simões, Wallisen Tadashi Hattori, Tânia Maria de Araújo

**Affiliations:** I Universidade Federal de Mato Grosso, Institute of Colletive Health – Cuiabá (MT), Brazil.; II Universidade Federal do Rio de Janeiro, Institute of Colletive Health Studies – Rio de Janeiro (RJ), Brazil.; III Universidade de São Paulo, School of Public Health – São Paulo (SP), Brazil.; IV Fundação Oswaldo Cruz, National School of Public Health, Department of Epidemiology – Rio de Janeiro (RJ), Brazil.; V Escola de Saúde Pública do Estado de Minas Gerais – Belo Horizonte (MG), Brazil.; VI Universidade Federal de Pernambuco – Recife (PE), Brazil.; VII Universidade do Estado da Bahia – Salvador (BA), Brazil.; VIII Fundação Oswaldo Cruz, René Rachou Institute, Center for Studies in Public Health and Aging – Belo Horizonte (MG), Brazil.; IX Universidade Federal de Minas School of Nursing, Department of Health Management – Belo Horizonte (MG), Brazil.; X Universidade Federal de Uberlândia, School of Medicine, Department of Collective Health – Uberlândia (MG), Brazil.; XI Universidade Estadual de Feira de Santana, Department of Health– Feira de Santana (BA), Brazil.

**Keywords:** Epidemiology, Health postgraduate programs, Teaching, Public health

## Abstract

**Objective:**

To present a critical analysis of the teaching of epidemiology in graduate programs in public health in Brazil.

**Methods:**

Descriptive study in two stages, based on a documentary survey (thematic axes of the courses in graduate programs evaluated by CAPES) and a survey with a convenience sample of faculty and students from programs in the field. The evaluation focused on graduate education in public health, aiming to critically examine the curricular proposals, pedagogical practices, and institutional conditions that shape epidemiology training at this level.

**Results:**

Important advances were identified in the teaching of epidemiology in graduate public health programs in Brazil, such as a significant number of courses offered, the consolidation of conceptual courses in academic programs, and the diversity and depth of thematic courses—particularly the topic of health services and systems, present in more than half of both academic and professional programs. Persistent challenges highlight the need to consolidate critical, consistent training that aligns with the contemporary demands of public health in the country and the Unified Health System (SUS).

**Conclusion:**

The reflections and actions outlined may help guide the next steps toward overcoming structures that perpetuate social inequalities and toward strengthening high-quality epidemiological training committed to transforming population health.

## INTRODUCTION

Epidemiology is a basic scientific discipline in the field of collective health (CH), responsible for generating knowledge, information, and technologies for the formulation and evaluation of policies for the promotion, prevention, and control of health problems^1^. In addition to its technical-scientific foundation, it carries the social and political commitment that characterizes CH. Therefore, the historical and conceptual transformations of this field must be incorporated into the training of professionals, faculty, and researchers in epidemiology^2^. Professional qualifications through CH training, both at the undergraduate and graduate levels, represent a strategic component for the implementation and improvement of public health policies and other intersectoral social policies. This relevance is expressed by the insertion of these professionals in decision-making and operational spaces at different levels of the system, working on the formulation, implementation, and evaluation of actions and programs. In this context, epidemiology training must transcend the mere transmission of technical content, incorporating a critical perspective that connects epidemiological knowledge with other areas of knowledge and concrete social demands. Such training must reaffirm its political and social commitment, guiding the application of knowledge to act on social determinants of health and promote equity, thus contributing to the transformation of the general health conditions of populations^3^.

Epidemiology training involves multiple and complex demands that are increasingly evident in light of the contemporary challenges facing health systems. Recent crises such as the COVID-19 pandemic, environmental disasters, and the reemergence of infectious diseases, combined with persistent social and territorial inequalities, reinforce the centrality of epidemiology in formulating rapid, effective, and ethically committed responses to the community^4,5^. This scenario has required professionals in the field not only technical and methodological expertise, but also analytical skills, intersectoral action, and an ethical and political commitment to CH.

The Brazilian Association of Public Health (Abrasco) has played a central role in the coordination between higher education institutions and the services of the Unified Health System (SUS), fostering dialogue and strengthening training opportunities in public health. In 2005, the Abrasco Epidemiology Commission developed the Fourth Strategic Plan, structured around three pillars: teaching, research, and policy. In the area dedicated to teaching, the document emphasized the importance of expanding access to scientific knowledge, keeping up with the accelerated renewal of the field, and modernizing pedagogical resources, including the incorporation of new technologies^6^.

Since the publication of the Fourth Strategic Plan, significant changes have marked both the technical and scientific development of epidemiology and the health demands and needs of the Brazilian population, intensified by the unprecedented health crisis of the COVID-19 pandemic. Given this new scenario, the evaluation of epidemiology teaching has become a fundamental step to support both the development of the Fifth Strategic Plan and its implementation over the next five years—a priority proposal of the Abrasco Epidemiology Commission for the coming years.

This scenario reinforces the importance of well-conducted evaluations regarding the diagnosis of epidemiology teaching and the identification of needs and areas of action within the current context of the health demands of the Brazilian population. To contribute to the dialogue on this topic, the Abrasco Epidemiology Commission 2022–2025, at the initiative of the Epidemiology Teaching Working Group, conducted a comprehensive national mapping of epidemiology teaching in undergraduate and graduate programs in CH in Brazil. The results of this initiative, presented at the II Workshop for the Development of the Fifth Strategic Plan for the Development of Epidemiology in Brazil (2025–2029), supported the development of this article.

The objective of this study was to critically analyze the curricular proposals, pedagogical practices, and institutional conditions that shape the teaching of epidemiology in stricto sensu graduate programs in CH in Brazil, on the basis of data collected through the mapping conducted by Abrasco within the scope of the Fifth Strategic Plan for the Development of Epidemiology in Brazil (2025–2029).

## METHODS

This was an exploratory study on the teaching of epidemiology in postgraduate programs (PPGs) in the CH field, structured in two stages. The first consisted of a documentary analysis, with data collection conducted between August and November 2023, using information available on the Sucupira Platform of the Coordination for the Improvement of Higher Education Personnel (CAPES), for the 2017–2020 evaluation period. Courses with epidemiology content offered in 54 academic and 41 professional programs were evaluated. The second stage involved an online survey of faculty and students enrolled in these programs.

In the documentary analysis stage, the CAPES Sucupira Platform, which provides annual public data on PPGs in CH, was the database consulted to obtain data such as syllabuses, course load, and course bibliography. Information on required and elective courses was collected, organized by thematic axes, according to the 2017–2020 four-year period. The courses were classified into 34 themes grouped into five axes (Supplementary Material 1). The courses were further categorized according to their specific themes, distinguishing those with an emphasis on epidemiology from those with specific themes, such as health, environment and work, or nutrition in public health, also considering the distribution by geographic region of the country and the year the course began.

In the second stage, a survey was conducted through an online questionnaire sent to graduate faculty and another specific questionnaire for students, distributed by the coordinators of each course. The form was sent to the official e-mail lists of all PPGs in CH registered on the Sucupira Platform, in addition to being distributed by the Abrasco Epidemiology Commission.

A convenience sample of 95 faculty members working in epidemiology courses and/or course coordinators was estimated, considering at least one respondent per course registered with CAPES. For students, the estimated participation of at least one student per course in each program was estimated. Among the 54 academic programs, there were 51 master's degrees and 38 doctorates, and among the 41 professional programs, 41 master's degrees and three doctorates were offered.

Data were collected between November 2023 and June 2024. Faculty members responsible for epidemiology courses in graduate programs were included in the study, with no minimum length of experience defined. For students, the inclusion criterion was being enrolled in the final year of the PPG (academic or professional), considering that this group has already completed the majority of their curricular training. Both questionnaires (faculty and student) included questions about sociodemographic characteristics—sex, race/skin color, education level—and the type of program and institution in which they were enrolled. The questionnaire was administered anonymously, ensuring the anonymity and confidentiality of the information collected. Regarding epidemiology teaching, respondents were asked about the perceived adequacy of the curriculum content offered for epidemiology training, as well as the pedagogical methods and strategies used, including the use of active methodologies—understood as student-centered approaches that place students as protagonists of the learning process through interactive activities, problem-solving, and practical application of knowledge.

The practice spaces and the relationships between disciplines and health policies and services were also assessed through the following questions, answered by faculty and students: "Are there activities focused on real-world health problems, directly related to public health policies or programs?"; and "Are there activities focused on real public health problems, based on some relationship with management spaces and/or health services?" Response options followed a Likert-type scale: never, rarely, occasionally, often, very often.

For faculty and students, open-ended questions were included to identify barriers and facilitators in the learning process in epidemiology courses, as well as difficulties in incorporating practical activities or their articulation with health services and policies. Closed-ended questions were analyzed on the basis of the absolute and relative frequency of responses. The essay responses were grouped by recurring themes and axes, considering barriers and facilitators as strata.

The project was approved by the Research Ethics Committee of the Institute of Studies in Collective Health at the Federal University of Rio de Janeiro, under No. 6.041.864. The informed consent form was made available on Google Forms for prior reading. Agreement was recorded by selecting the "Yes" option. Selecting "No" automatically closed the form.

## RESULTS

**Figure 1:**
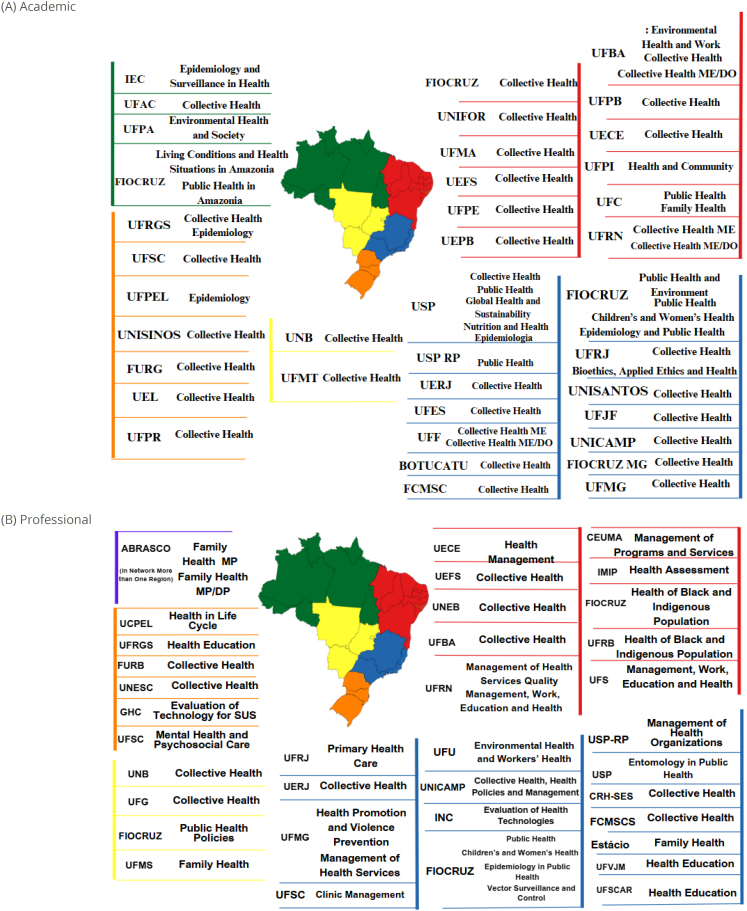
Higher education institutions offering (A) academic and (B) professional postgraduate courses at the master's and/or doctoral level in collective health in Brazil, 2021.

Of the 54 academic and 41 professional programs (Figure 1), the highest concentration was found in the Southeast (23; 44.2% and 18; 43.9%) and Northeast (16; 43.2% and 12; 32.4%) regions, followed by the South (8; 13.5% and 6; 14.6%), respectively. Two professional programs were offered in a network by institutions in different regions. Regarding the thematic distribution by region, the Southeast showed the greatest diversity of focuses, followed by the Northeast, concentrating all three professional master's degrees in epidemiology (Table 1).

IEC: Evandro Chagas Institute UFAC: Federal University of Acre; UFPA: Federal University of Pará; FIOCRUZ: Oswaldo Cruz Foundation; Unifor: University of Fortaleza; UFMA: Federal University of Maranhão; UEFS: State University of Feira de Santana; UEPE: State University of Pernambuco; UEPB: State University of Paraíba; UFBA: Federal University of Bahia; UFPB: Federal University of Paraíba; UECE: State University of Ceara; UFPI: Federal University of Piauí; UFC: Federal University of Ceara; UFRN: Federal University of Rio Grande do Norte; UFRGS: Federal University of Rio Grande do Sul; UFSC: Federal University of Santa Catarina; UFPel: Federal University of Pelotas; Unisinos: University of Vale do Rio dos Sinos; Furg: Federal University of Rio Grande; UEL: State University of Londrina; UFPR: Federal University of Paraná; UnB: University of Brasília; UFMT: Federal University of Mato Grosso; USP: University of São Paulo; USP RP: University of São Paulo — Ribeirão Preto Campus; UERJ: Rio de Janeiro State University; UFES: Federal University of Espírito Santo; UFF: Fluminense Federal University; FCMSC: Santa Casa School of Medical Sciences; UFRJ: Federal University of Rio de Janeiro; Unisantos: Catholic University of Santos; UFJF: Federal University of Juiz de Fora; Unicamp: State University of Campinas; UFMG: Federal University of Minas Gerais; Abrasco: Brazilian Association of Public Health; UCPel: Catholic University of Pelotas; Furb: Regional University of Blumenau; Unesc: University of the Extreme South of Santa Catarina; GHC: Conceição Hospital Group; SUS: Unified Health System; UFG: Federal University of Goiás; UFMS: Federal University of Mato Grosso do Sul; UFRB: Federal University of Recôncavo da Bahia; UFS: Federal University of Sergipe; UFU: Federal University of Uberlândia; INC: National Institute of Cardiology; CRH-SES: Human Resources Coordination of the State Department of Health; FCMSCS: Faculty of Medical Sciences of São Carlos; UFVJM: Federal University of the Jequitinhonha and Mucuri Valleys; UFSCar: Federal University of São Carlos; Uneb: State University of Bahia; Ceuma: Ceuma University; IMIP: Professor Fernando Figueira Institute of Integral Medicine.

**Table 1:** Distribution of postgraduate programs by major thematic group, according to geographic region and year of commencement. Brazil, 2021.

		Total n (%)	Collective/public n (%)	Epidemiology n (%)	With focus (such as child health, environment…) n (%)
		Academic programs			
		54 (100.0)	38 (100.0)	5 (100.0)	11 (100.0)
**Region**					
	North	5 (9.6)	1 (2.6)	1 (20.0)	3 (27.3)
	Northeast	16 (28.8)	13 (34.2)	-	3 (27.3)
	Central-West	2 (3.8)	2 (5.3)	-	-
	South	8 (13.5)	6 (15.8)	2 (40.0)	-
	Southeast	23 (44.2)	16 (42.1)	2 (40.0)	5 (45.4)
**Year started**					
	1970–2009	19 (35.2)	15 (39.5)	1 (20.0)	3 (27.3)
	2010–2016	29 (53.7)	19 (50.0)	3 (60.0)	7 (63.6)
	2017–2021	6 (11.1)	4 (10.5)	1 (20.0)	1 (9.1)
		Professional programs			
		41 (100.0)	13 (100.0)	2 (100.0)	26 (100.0)
**Region**					
	North	-	-	-	-
	Northeast	11 (26.8)	4 (30.8)	-	7 (26.9)
	Central-West	4 (9.7)	2 (15.4)	-	2 (7.7)
	South	6 (14.6)	2 (15.4)	-	4 (15.4)
	Southeast	18 (43.9)	5 (38.5)	2 (100.0)	11 (42.3)
	Network (more than one region)	2 (4.9)	-	-	2 (7.7)
**Year started**					
	1970–2009	6 (14.6)	4 (30.8)	-	2 (7.9)
	2010–2016	27 (65.8)	8 (61.5)	1 (50.0)	17 (65.3)
	2017–2021	8 (19.5)	1 (7.7)	1 (50.0)	7 (26.9)

The offering of 1,059 courses was identified in some year during the 2017–2020 quadrennium. The absence of courses with epidemiology content was observed in only three programs. All academic programs offered at least one conceptual course in the area, while in professional programs this offering was less frequent (61.0%). Courses focused on data collection, processing, and analysis were also more frequent in academic programs (92.6%) than in professional programs (68.3%). Courses with surveillance content were less frequent, present in only 33.3% of academic programs and 26.3% of professional programs. Courses focused on health services and systems were present in more than half of academic and professional programs (55.5% and 53.6%, respectively). Finally, academic programs stood out for offering thematic courses (90.7%), in contrast to professional programs, where only 41.5% offered this type of course (Table 2).

**Table 2:** Distribution of academic and professional postgraduate programs according to the offering of epidemiology courses by thematic axis. Brazil, 2021.

	Total n (%)	Collective/public n (%)	Epidemiology n (%)	With focus (such as child health, environment…) n (%)
	54 (100.0)	38 (100.0)	5 (100.0)	11 (100.0)
Conceptual in the field	54 (100.0)	38 (100.0)	5 (100.0)	10 (90.9)
Related to data collection. processing and analysis	50 (92.6)	36 (94.7)	4 (80.0)	10 (90.9)
Surveillance	18 (33.3)	14 (36.8)	1 (20.0)	3 (27.3)
Focused on health services and systems	30 (55.5)	19 (50.0)	4 (80.0)	6 (54.5)
Thematics	49 (90.7)	31 (81.6)	5 (100.0)	11 (100.0)
	Professional programs			
	41 (100.0)	13 (100.0)	3 (100.0)	25 (100.0)
Conceptual in the field	25 (61.0)	11 (84.6)	2 (66.7)	12 (48.0)
Related to data collection. processing and analysis	28 (68.3)	10 (76.9)	2 (66.7)	15 (60.0)
Surveillance	11 (26.8)	5 (38.5)	2 (66.7)	4 (16.0)
Focused on health services and systems	22 (53.6)	12 (92.3)	1 (33.3)	11 (44.0)
Thematics	17 (41.5)	6 (46.1)	3 (100.0)	8 (32.0)

**Figure 2:**
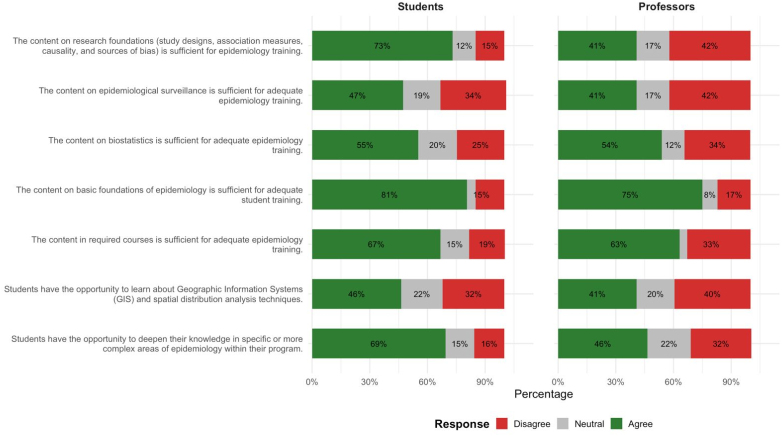
Distribution of reports from faculty and students regarding the content taught in epidemiology courses in postgraduate courses in public health. Brazil, 2021.

Most faculty and students agreed that the core and required content was sufficient for adequate training in epidemiology. Significant differences were observed regarding epidemiological research content and thematic in-depth study: while 73% of students considered the research content adequate, only 41% of faculty agreed with this assertion. In turn, 69% of students positively evaluated the possibilities for further study in the field, while only 46% of faculty agreed with this statement. Furthermore, approximately 40% of faculty considered that the content on the fundamentals of epidemiological research and epidemiological surveillance was insufficient for adequate training in epidemiology (Figure 2).

**Figure 3:**
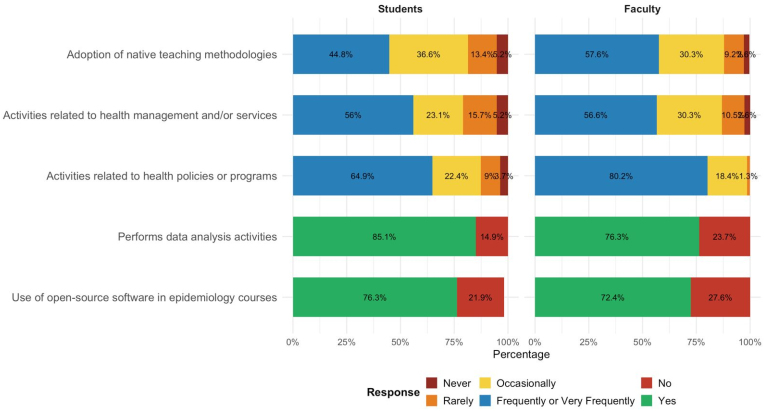
Distribution of teaching methods and software use by faculty working in epidemiology courses in postgraduate and undergraduate programs in collective health. Brazil, 2024.

The frequent or very frequent use of active methodologies in teaching was reported by 58% of faculty and 45% of students. Data analysis activities and the use of free software in teaching were widely reported by both categories (76 and 72% of faculty and 85 and 76% of students, respectively). Activities related to real-world healthcare problems and public policies were reported as frequently or very frequently by over 80% of faculty and approximately 65% of students. Activities directly related to management or health services were reported as frequent or very frequent by 57% of faculty and 56% of students (Figure 3), indicating similar participation between the two groups.

When asked about the barriers that compromise epidemiology teaching in graduate programs, study participants cited deficiencies in students’ prior knowledge as the most frequent, especially in mathematics, scientific writing, and basic training in epidemiology and biostatistics. The heterogeneity of prior training hinders the understanding of fundamental concepts and the mastery of more complex analyses. Institutional limitations were also reported, such as a lack of teaching resources and insufficient course loads for faculty to cover essential content.

The main facilitators of learning in epidemiology mentioned by faculty were access to infrastructure and institutional resources. The use of free software and statistical packages, good physical infrastructure (computer labs, classrooms, libraries), and the availability of local data and educational technologies stand out. Other facilitating strategies cited were the adoption of active teaching methods, hands-on activities, and flexibility in class formats, including in-person, remote, or hybrid modalities (data not presented in tables or charts).

From the perspective of graduate students, the main barriers to learning in epidemiology courses related to the traditional teaching methods adopted by faculty, the predominance of lecture methods, and the use of assessment strategies aligned poorly with the educational process. Difficulties related to limited workload for in-depth content coverage and some faculty members’ resistance to incorporating new technologies and teaching methodologies were also mentioned. In contrast, the main facilitators of learning were the faculty members’ training and teaching skills, the adoption of active and participatory methodologies, and the use of practical examples linked to real-life public health contexts. These elements are valued for fostering greater student engagement, better understanding of content, and a connection between theory and practice, reinforcing the importance of more dynamic, contextualized, and student-centered pedagogical practices (data not presented in tables or charts).

## DISCUSSION

The present study showed that there was a greater concentration of PPGs in CH in the Southeast and Northeast regions, with greater thematic diversity in the Southeast. The curriculum analysis revealed that, although most programs offered conceptual and methodological courses, there was less content focused on health surveillance, especially in professional programs. Furthermore, challenges in training for faculty and students related to the heterogeneity of prior training, insufficient course load, and traditional teaching methods were highlighted, while facilitating factors were identified, such as the use of free software and the incorporation of activities focused on problems directly related to health policies or programs.

A greater offering of epidemiology courses was also observed in academic PPGs compared to professional PPGs, demonstrating its importance as a structuring axis of the field. Historically, epidemiology teaching in Brazil has focused on public health^4,7^. Currently, epidemiology is fundamental to health surveillance, coordinating responses to public health emergencies, and other regional and global priorities^4,8,9^.

It is noteworthy that less than half of academic and professional programs offered courses focused on surveillance, and about half of them included courses related to health services and systems, indicating that technical and political training in this area remained limited. The Fifth Strategic Plan for the Development of Epidemiology in Brazil (2025–2029) proposes to restore the centrality of surveillance in epidemiology education and strengthen its connection with health services and intersectoral practices.

According to Lau et al.^10^, epidemiology training should include, in addition to analytical methods, practical application in real-world settings, communication of results, and integration with services. The authors proposed a basic framework for epidemiology training aimed at maintaining this field of knowledge as fundamental for addressing new public health challenges. The proposal considered that the applied epidemiology area should include not only study designs and sophisticated statistical analyses, but also aspects of health surveillance, primary data collection, manipulation and analysis of data collected in routine services, in addition to robust training in descriptive and field epidemiology.

Furthermore, approximately 40% of faculty considered that the content focused on the fundamentals of epidemiological research and surveillance being insufficient to ensure adequate training in the field, revealing an important critical perception of structural gaps in program curricula. This finding directly aligns with Aquino's^11^ reflections, which appear to remain relevant. The author pointed out that, despite the expansion of graduate programs and the intensification of scientific production, a training model excessively focused on theoretical disciplines and disconnected from concrete investigative practice still persists. To overcome this situation, Aquino^11^ advocates the adoption of pedagogical strategies more integrated with research that involve students in collective experiences, such as research consortia, and favor training in applied, socially relevant contexts connected to the needs of SUS. The results presented here suggest that such strategies are not yet widely incorporated, especially in professional programs, and highlight the need for a more consistent institutional repositioning focused on critical, applied training that is committed to the country's health reality.

As highlighted in the Fifth Strategic Plan for the Development of Epidemiology in Brazil (2025–2029), it is necessary to overcome fragmented curricula that are disconnected from the needs of SUS in training in CH. This criticism is reinforced by Barata^3^, who points out that postgraduate education in the field still lacks greater coordination with the demands of the health system. Similarly, Werler et al.^12^, when discussing the teaching of epidemiology internationally, advocate the development of more integrated, critical curricula focused on the application of knowledge in real-world contexts, in the face of new scientific and social challenges. In the present study, despite the broad presence of conceptual and thematic disciplines, especially in academic programs, this alone does not guarantee critical training integrated with the needs of the population and health systems. Therefore, the critical and technical training of epidemiologists must be guided not only by methodological excellence but also by the ability to act and adapt in complex scenarios and changing social realities. The increasing incidence of natural disasters and health emergencies, for example, has expanded the scope of epidemiology, requiring professionals trained to deal with highly complex scenarios and collective risk. The review by Liu et al.^13^ shows that disaster epidemiology has been consolidating itself as a strategic field, with a significant increase in scientific production and a focus on topics such as post-disaster surveillance, health risk assessment, rapid response, and the use of analytical tools to support decision-making. These advances reinforce the need to incorporate specific competencies into epidemiology training.

Epidemiology teaching in Brazil has undergone substantial transformations, especially regarding the adoption of active methodologies that seek to make the learning process more dynamic, participatory, and student-centered. The results showed that practical, territorially based activities have been incorporated; approximately 80% of faculty reported incorporating classroom activities focused on real-world health problems directly related to health policies or programs. As advocated in the Fifth Strategic Plan for the Development of Epidemiology in Brazil (2025–2029), these activities can bring teaching closer to the real needs of SUS and vulnerable populations.

Lima et al.^14^, reporting on their experience teaching epidemiology, point to the use of active methodologies such as problem-based learning, flipped classrooms, and mind maps in undergraduate courses. The authors found significant gains in student engagement, content retention, and strengthening of the bond between faculty and students. However, they also highlight structural limitations, such as limited workloads and rigid theoretical content, which hinder the full application of these methodologies. These findings reinforce the need to rethink curricula, promoting greater pedagogical flexibility and valuing critical and participatory approaches.

The concentration of PPGs in the Southeast and Northeast regions highlights the disparity within Brazil. The Southeast region still has the greatest thematic diversity in epidemiology education, reinforcing regional inequalities in specialized training opportunities, given that undergraduate programs in CH are concentrated in other regions. The majority of undergraduate programs in CH between 2008 and 2014 were concentrated in the Northeast region and are currently concentrated in the North region due to the opening of programs and universities, particularly public universities, which account for 68.5% of these programs^15^.

Finally, the need for more robust development of scientific communication is highlighted, as it disseminates accurate and reliable information to the public. This demand was evident during the COVID-19 pandemic but has been reinforced to a lesser extent since the pandemic, because of topics related to vaccination, climate change, and emerging and re-emerging diseases. This strategy will help train epidemiologists capable of communicating with different audiences and combating misinformation, including on complex topics such as vaccination, climate change, and emerging diseases.

This study focused exclusively on stricto sensu PPGs in the collective health area of CAPES, aiming to analyze in depth the inclusion of epidemiology in this specific field of training. It is recognized, however, that epidemiology also plays a central role in undergraduate health programs and in postgraduate programs in other areas of assessment, such as medicine I and II, nursing, and nutrition, among others, as well as courses in other training modalities. The delimitation adopted was a necessary methodological approach to ensure greater rigor and depth in the analysis, although it does not exhaust the debate on the challenges and potential of teaching epidemiology in the broader context of health education. On the other hand, this is the first initiative to outline the profile of the programs and the perspective of PPG faculty and students regarding the teaching of epidemiology. The findings of this study indicate important advances, such as the presence of structuring disciplines, the adoption of teaching strategies with activities directly related to health policies or programs, and the incorporation of active methodologies. They also highlight significant gaps in critical and applied training in epidemiology, especially in professional programs.

Given this scenario, it is necessary to strengthen the surveillance and applied research axis in curricula and to value pedagogical strategies integrated with health services. Greater investment in teaching infrastructure, faculty support, and interprogram coordination is also recommended to encourage collaborative experiences, such as networked learning. Finally, the importance of incorporating content related to scientific communication, data analysis in real-world contexts, and addressing regional inequalities is emphasized as essential dimensions for the training of epidemiologists capable of responding to current health challenges in Brazil.

## Data Availability

The dataset supporting the findings of this study is not publicly available.
