# Supplementary material for: Critical mapping of epidemiology training in graduate programs in collective health in Brazil: challenges and perspectives
Source: Rev Bras Epidemiol. 2025 Nov 17;28(Suppl 1):e250004supl1. doi: 10.1590/1980-549720250004.supl.1 (PMC12622985; doi:10.1590/1980-549720250004.supl.1)
Supplement: Material Suplementar 1 [file 1980-5497-rbepid-28-suppl1-e250004supl1-Suppl01.pdf]

Quadro suplementar: Eixos e temas considerados na análise das disciplinas dos cursos de pós-graduação acadêmicos e profissionais em Saúde Coletiva. Brasil, 2021.

Eixo	Temas abordados nas disciplinas
**Conceituais da área**	Epidemiologia avançada
	Epidemiologia básica ou geral
	Desenhos de Estudos Epidemiológicos
	Epidemiologia social
	Geoprocessamento e Saúde
	Epidemiologia e metodologia de pesquisa
**Relacionadas à coleta, processamento e análise de dados**	Bioestatística (avançada ou específica)
	Bioestatística (geral ou introdutória)
	Construção e validação de instrumentos
	Epidemiologia de campo
	Epidemiologia e Análise de Dados
	Matemática aplicada à saúde
	Informática Aplicada à Epidemiologia
**Voltados à serviços e sistemas de saúde**	Epidemiologia e Serviços de Saúde
	Epidemiologia e Sistemas de informação em saúde
**Vigilância em saúde**	Vigilâncias ambiental, sanitária e epidemiológica
	Vigilâncias específicas como doenças transmissíveis e saúde bucal, do trabalhador
**Temáticos**	Epidemiologia e saúde ambiental
	Epidemiologia Clínica
	Epidemiologia e Atividade Física
	Epidemiologia e Câncer
	Epidemiologia e Controle de infecções hospitalares
	Epidemiologia e Doenças Não Transmissíveis
	Epidemiologia e Doenças Transmissíveis
	Epidemiologia e Envelhecimento
	Epidemiologia das Diversidades Étnico-Raciais
	Epidemiologia e Saúde Bucal
	Epidemiologia e Saúde da família, da Mulher/Criança/Adolescente
	Epidemiologia e Saúde Mental
	Epidemiologia e Violência
	Epidemiologia Molecular, Genética e Infecções Hospitalares
	Epidemiologia Nutricional
	Epidemiologia, Saúde e Trabalho<br>Farmacoepidemiologia
	Epidemiologia e qualidade de vida
