# Supplementary material for: Critical mapping of epidemiology training in graduate programs in collective health in Brazil: challenges and perspectives
Source: Rev Bras Epidemiol. 2025 Nov 17;28(Suppl 1):e250004supl1. doi: 10.1590/1980-549720250004.supl.1 (PMC12622985; doi:10.1590/1980-549720250004.supl.1)
Supplement: Translation

# Translation: Mapeamento crítico da formação em epidemiologia nos programas de pós-graduação em Saúde Coletiva no Brasil: desafios e perspectivas

**DOI:** 10.1590/1980-549720250004.supl.1.2

**Authors:** Ana Paula Muraro, Amanda de Moura Souza, Cassia Maria Buchalla, Enirtes Caetano Prates Melo, Gerusa Gibson, João André Tavares Álvares da Silva, Lívia Teixeira de Souza Maia, Maria Aparecida Araújo Figueiredo, Sérgio Viana Peixoto, Taynãna César Simões, Wallisen Tadashi Hattori, Tânia Maria de Araújo

**Affiliations:** I Universidade Federal de Mato Grosso, Instituto de Saúde Coletiva – Cuiabá (MT), Brasil.; II Universidade Federal do Rio de Janeiro, Instituto de Estudos de Saúde Coletiva – Rio de Janeiro (RJ), Brasil.; III Universidade de São Paulo, Faculdade de Saúde Pública – São Paulo (SP), Brasil.; IV Fundação Oswaldo Cruz, Escola Nacional de Saúde Pública, Departamento de Epidemiologia – Rio de Janeiro (RJ), Brazil.; V Escola de Saúde Pública do Estado de Minas Gerais – Belo Horizonte (MG), Brasil.; VI Universidade Federal de Pernambuco – Recife (PE), Brasil.; VII Universidade do Estado da Bahia – Salvador (BA), Brasil.; VIII Fundação Oswaldo Cruz, Instituto René Rachou, Núcleo de Estudos em Saúde Pública e Envelhecimento – Belo Horizonte (MG), Brasil.; IX Universidade Federal de Minas Gerais, Escola de Enfermagem, Departamento de Gestão em Saúde – Belo Horizonte (MG), Brasil.; X Universidade Federal de Uberlândia, Faculdade de Medicina, Departamento de Saúde Coletiva – Uberlândia (MG), Brasil.; XI Universidade Estadual de Feira de Santana, Departamento de Saúde – Feira de Santana (BA), Brazil.

**Keywords:** Epidemiologia, Programas de pós-graduação em saúde, Ensino, Saúde pública

## Abstract

**Objetivo:**

Apresentar uma análise crítica do ensino de epidemiologia nos programas de pós-graduação em Saúde Coletiva no Brasil.

**Métodos:**

Estudo descritivo com duas etapas com base em levantamento documental (eixos temáticos das disciplinas dos programas de pós-graduação avaliados pela Coordenação de Aperfeiçoamento de Pessoal de Nível Superior) e inquérito com amostra de conveniência de docentes e discentes dos programas da área. Optou-se por focalizar a avaliação na pós-graduação em Saúde Coletiva, com o objetivo de examinar criticamente as propostas curriculares, as práticas pedagógicas e as condições institucionais que conformam a formação da epidemiologia nesse nível.

**Resultados:**

Identificaram-se avanços importantes no ensino de epidemiologia na pós-graduação em Saúde Coletiva no Brasil, como o expressivo número de disciplinas nos programas, a consolidação de disciplinas conceituais nos programas acadêmicos, a diversidade e o aprofundamento de disciplinas temáticas, destacando-se o tema serviços e sistemas de saúde, presente em mais da metade dos programas acadêmicos e profissionais. Os desafios persistentes apontam para a consolidação de uma formação crítica alinhada às demandas contemporâneas da saúde coletiva no país e do Sistema Único de Saúde.

**Conclusão:**

As reflexões e ações apontadas podem contribuir para orientar os próximos passos para superar estruturas que perpetuam desigualdades sociais e fortalecer uma formação epidemiológica qualificada e comprometida com a transformação da saúde das populações.

## INTRODUÇÃO

A epidemiologia é uma disciplina científica básica do campo da saúde coletiva (SC) responsável por gerar conhecimentos, informações e tecnologias para a formulação e avaliação das políticas de promoção, prevenção e controle dos problemas de saúde^1^. Além de seu fundamento técnico-científico, ela carrega o compromisso social e político que caracteriza a SC. Assim, as transformações históricas e conceituais desse campo devem ser incorporadas à formação de profissionais, docentes e pesquisadores em epidemiologia^2^.

A qualificação de profissionais por meio da formação em SC, tanto na graduação quanto na pós-graduação, representa um componente estratégico para a efetivação e o aprimoramento de políticas públicas de saúde e outras políticas sociais intersetoriais. Essa relevância expressa-se pela inserção desses profissionais em espaços decisórios e operacionais nos diferentes níveis do sistema, atuando na formulação, implementação e avaliação de ações e programas. Nesse contexto, a formação em epidemiologia deve transcender a mera transmissão de conteúdos técnicos, incorporando uma perspectiva crítica que articule o conhecimento epidemiológico a outras áreas do saber e às demandas sociais concretas. Tal formação deve reafirmar seu compromisso político e social, orientando a aplicação do conhecimento para atuar sobre determinantes sociais da saúde e promover equidade, contribuindo assim para a transformação das condições gerais de saúde das populações^3^.

A formação em epidemiologia envolve exigências múltiplas e complexas cada vez mais evidentes diante dos desafios contemporâneos enfrentados pelos sistemas de saúde. Crises recentes — como a pandemia de COVID-19, desastres ambientais, reemergência de doenças infecciosas —, combinadas às persistentes desigualdades sociais e territoriais, reforçam a centralidade da epidemiologia na formulação de respostas rápidas, eficazes e eticamente comprometidas com as coletividades^4,5^. Tal cenário tem exigido dos profissionais da área não apenas domínio técnico-metodológico, mas também capacidade analítica, atuação intersetorial e compromisso ético-político com a SC.

A Associação Brasileira de Saúde Coletiva (Abrasco) tem desempenhado papel central na articulação entre instituições de ensino superior e os serviços do Sistema Único de Saúde (SUS), favorecendo o diálogo e o fortalecimento das possibilidades de formação em SC. Em 2005, a Comissão de Epidemiologia da Abrasco elaborou o IV Plano Diretor de Desenvolvimento da Epidemiologia, estruturado em três eixos — ensino, pesquisa e políticas. No eixo dedicado ao ensino, o documento enfatizou a importância de se ampliar o acesso ao conhecimento científico, acompanhar a renovação acelerada da área e modernizar os recursos pedagógicos, incluindo a incorporação de novas tecnologias^6^.

Desde a publicação do IV Plano, mudanças expressivas marcaram tanto o desenvolvimento técnico-científico da epidemiologia quanto as demandas e necessidades em saúde da população brasileira, intensificadas pela vivência de uma crise sanitária sem precedentes, como foi a pandemia de COVID-19. Diante desse novo cenário, a avaliação do ensino da epidemiologia se tornou uma etapa fundamental tanto para subsidiar a elaboração do V Plano Diretor quanto para sua implementação no próximo quinquênio — uma proposta prioritária da Comissão de Epidemiologia da Abrasco para os próximos anos.

Esse cenário reforça a relevância de avaliações bem conduzidas no que se refere ao diagnóstico do ensino de epidemiologia e ao levantamento de necessidades e campos de atuação no contexto atual das demandas em saúde da população brasileira. Com o objetivo de colaborar com o diálogo sobre a temática, a Comissão de Epidemiologia da Abrasco 2022–2025, por iniciativa do Grupo de Trabalho de Ensino de Epidemiologia, realizou um amplo mapeamento nacional do ensino de epidemiologia nos cursos de graduação e pós-graduação em Saúde Coletiva no Brasil. Os resultados dessa iniciativa, apresentados na II Oficina para a elaboração do V Plano Diretor para o Desenvolvimento da Epidemiologia no Brasil (2025–2029), subsidiaram a elaboração deste artigo.

O objetivo deste estudo é analisar criticamente as propostas curriculares, as práticas pedagógicas e as condições institucionais que conformam o ensino de epidemiologia nos programas de pós-graduação *stricto sensu* em Saúde Coletiva no Brasil, com base nos dados coletados, por meio do mapeamento conduzido pela Abrasco no âmbito do V Plano Diretor para o Desenvolvimento da Epidemiologia no Brasil (2025–2029).

## Métodos

Trata-se de uma pesquisa exploratória sobre o ensino de epidemiologia nos programas de pós-graduação (PPG) da área de SC estruturada em duas etapas. A primeira consistiu em uma análise documental, com coleta de dados realizada entre agosto e novembro de 2023, por meio das informações disponíveis na Plataforma Sucupira da Coordenação de Aperfeiçoamento de Pessoal de Nível Superior (Capes), referente ao quadriênio de avaliação 2017–2020. Foram avaliadas as disciplinas com conteúdo de epidemiologia, ofertadas nos 54 programas acadêmicos e 41 profissionais. A segunda etapa compreendeu um inquérito *online* com docentes e discentes matriculados nesses cursos.

Na etapa da análise documental, a Plataforma Sucupira da Capes, que disponibiliza dados públicos anuais dos PPG em Saúde Coletiva, foi a base consultada para obtenção de dados como ementa, carga horária e bibliografia das disciplinas. Foram coletadas as informações sobre disciplinas obrigatórias e optativas, organizadas por eixos temáticos, com base no quadriênio 2017–2020. As disciplinas foram classificadas em 34 temáticas agrupadas em cinco eixos (Material Suplementar 1). Os cursos foram ainda categorizados segundo suas temáticas específicas, distinguindo-se aqueles com ênfase em epidemiologia daqueles com temáticas específicas, como saúde, ambiente e trabalho, ou nutrição em saúde pública, considerando também a distribuição por região geográfica do país e o ano de início do curso.

Na segunda etapa, foi realizado um inquérito por meio de questionário *online* enviado para os docentes dos cursos de pós-graduação e outro questionário específico para os discentes, divulgado pela coordenação de cada curso. O formulário foi enviado para a lista de *e-mails* oficiais de todos os PPG em SC cadastrados na Plataforma Sucupira, além de ter sido divulgado pela Comissão de Epidemiologia da Abrasco.

Foi estimada uma amostra de conveniência de 95 docentes atuantes em disciplinas de epidemiologia e/ou coordenadores dos cursos, considerando pelo menos um respondente por curso cadastrado na Capes. Para os discentes, estimou-se a participação de pelo menos um estudante por curso de cada programa. Entre os 54 programas acadêmicos, havia 51 mestrados e 38 doutorados, e entre os 41 programas profissionais, ofertavam-se 41 mestrados e três doutorados.

Os dados foram coletados entre novembro de 2023 e junho de 2024. Foram incluídos no estudo docentes responsáveis por disciplinas na área de epidemiologia nos cursos de pós-graduação, sem definição de tempo mínimo de atuação. Para os discentes, foi considerado como critério de inclusão estar matriculado no último ano do PPG (acadêmico ou profissional), considerando-se que esse grupo já vivenciou a maior parte da formação curricular.

Ambos os questionários (docentes e discentes) incluíram questões sobre características sociodemográficas — gênero, raça/cor, escolaridade — e o tipo de curso e instituição ao qual era vinculado/matriculado. O questionário foi feito sem a identificação dos respondentes, garantindo o anonimato e a confidencialidade das informações coletadas. Sobre o ensino de epidemiologia, questionou-se a percepção de adequação dos conteúdos da matriz curricular ofertada para a formação em epidemiologia, bem como métodos e estratégias pedagógicas aplicados, incluindo o uso de metodologias ativas — entendidas como abordagens centradas no estudante, que o colocam como protagonista do processo de aprendizagem, por meio de atividades interativas, resolução de problemas e aplicação prática dos conhecimentos.

Avaliaram-se também os espaços de práticas e as relações entre as disciplinas e as políticas e os serviços de saúde, mediante as seguintes questões, respondidas por docentes e discentes: "Estão presentes atividades voltadas para os problemas reais da SC, relacionados diretamente com políticas ou programas de saúde pública?"; e "Estão presentes atividades voltadas para os problemas reais da saúde coletiva, a partir de alguma relação com os espaços de gestão e/ou serviços de saúde?". As opções de resposta seguiram a escala tipo Likert: nunca, raramente, ocasionalmente, frequentemente, muito frequentemente.

Para docentes e discentes, foram incluídas questões abertas voltadas à identificação de barreiras e de facilitadores no processo de aprendizagem nas disciplinas de epidemiologia, bem como dificuldades para a incorporação de atividades práticas ou sua articulação com os serviços e políticas de saúde. As questões fechadas foram analisadas com base na frequência absoluta e relativa das respostas. As respostas dissertativas foram agrupadas por temas e eixos recorrentes, considerando como estratos as barreiras e os facilitadores.

O projeto foi aprovado pelo Comitê de Ética em Pesquisa do Instituto de Estudos em Saúde Coletiva da Universidade Federal do Rio de Janeiro, sob parecer de número 6.041.864. O termo de consentimento livre e esclarecido foi disponibilizado no Google Forms, para leitura prévia. A concordância foi registrada ao selecionar-se a opção "sim". A opção "não" encerrava automaticamente o formulário.

## RESULTADOS

Dos 54 programas acadêmicos e 41 profissionais (Figura 1), verificou-se maior concentração nas regiões Sudeste (23; 44,2% e 18; 43,9%) e Nordeste (16; 43,2% e 12; 32,4%), seguidas da Região Sul (8; 13,5% e 6; 14,6%), respectivamente. Dois programas profissionais eram ofertados em rede por instituições de diferentes regiões. Em relação à distribuição temática por região, o sudeste apresentou a maior diversidade de enfoques, seguido da Região Nordeste, concentrando todos os três mestrados profissionais de epidemiologia (Tabela 1).

**Figura 1:** Instituições de ensino superior com cursos de pós-graduação em nível de mestrado e/ou doutorado (A) acadêmico e (B) profissionais em Saúde Coletiva no Brasil, 2021.

IEC: Instituto Evandro Chagas; UFAC: Universidade Federal do Acre; UFPA: Universidade Federal do Pará; Fiocruz: Fundação Oswaldo Cruz; Unifor: Universidade de Fortaleza; UFMA: Universidade Federal do Maranhão; UEFS: Universidade Estadual de Feira de Santana; UEPE: Universidade Estadual de Pernambuco; UEPB: Universidade Estadual da Paraíba; UFBA: Universidade Federal da Bahia; UFPB: Universidade Federal da Paraíba; UECE: Universidade Estadual do Ceará; UFPI: Universidade Federal do Piauí; UFC: Universidade Federal do Ceará; UFRN: Universidade Federal do Rio Grande do Norte; UFRGS: Universidade Federal do Rio Grande do Sul; UFSC: Universidade Federal de Santa Catarina; UFPel: Universidade Federal de Pelotas; Unisinos: Universidade do Vale do Rio dos Sinos; Furg: Universidade Federal do Rio Grande; UEL: Universidade Estadual de Londrina; UFPR: Universidade Federal do Paraná; UnB: Universidade de Brasília; UFMT: Universidade Federal de Mato Grosso; USP: Universidade de São Paulo; USP RP: Universidade de São Paulo — *Campus* de Ribeirão Preto; UERJ: Universidade do Estado do Rio de Janeiro; UFES: Universidade Federal do Espírito Santo; UFF: Universidade Federal Fluminense; FCMSC: Faculdade de Ciências Médicas de Santa Casa; UFRJ: Universidade Federal do Rio de Janeiro; Unisantos: Universidade Católica de Santos; UFJF: Universidade Federal de Juiz de Fora; Unicamp: Universidade Estadual de Campinas; UFMG: Universidade Federal de Minas Gerais; Abrasco: Associação Brasileira de Saúde Coletiva; UCPel: Universidade Católica de Pelotas; Furb: Universidade Regional de Blumenau; Unesc: Universidade do Extremo Sul Catarinense; GHC: Grupo Hospitalar Conceição; SUS: Sistema Único de Saúde; UFG: Universidade Federal de Goiás; UFMS: Universidade Federal de Mato Grosso do Sul; UFRB: Universidade Federal do Recôncavo da Bahia; UFS: Universidade Federal de Sergipe; UFU: Universidade Federal de Uberlândia; INC: Instituto Nacional de Cardiologia; CRH-SES: Coordenação de Recursos Humanos da Secretaria Estadual de Saúde; FCMSCS: Faculdade de Ciências Médicas de São Carlos; UFVJM: Universidade Federal dos Vales do Jequitinhonha e Mucuri; UFSCar: Universidade Federal de São Carlos; Uneb: Universidade do Estado da Bahia; Ceuma: Universidade Ceuma; IMIP: Instituto de Medicina Integral Professor Fernando Figueira.

**Tabela 1:** Distribuição dos programas de pós-graduação por grande grupo temático, segundo regiões geográficas e ano de início. Brasil, 2021.

		Total N (%)	Saúde coletiva/pública n (%)	Epidemiologia n (%)	Com foco (como saúde da criança, ambiente…) n (%)
		Programas acadêmicos			
		54 (100,0)	38 (100,0)	5 (100,0)	11 (100,0)
**Região**					
	Norte	5 (9,6)	1 (2,6)	1 (20,0)	3 (27,3)
	Nordeste	16 (28,8)	13 (34,2)	-	3 (27,3)
	Centro-Oeste	2 (3,8)	2 (5,3)	-	-
	Sul	8 (13,5)	6 (15,8)	2 (40,0)	-
	Sudeste	23 (44,2)	16 (42,1)	2 (40,0)	5 (45,4)
**Ano de início**					
	1970–2009	19 (35,2)	15 (39,5)	1 (20,0)	3 (27,3)
	2010–2016	29 (53,7)	19 (50,0)	3 (60,0)	7 (63,6)
	2017–2021	6 (11,1)	4 (10,5)	1 (20,0)	1 (9,1)
		Programas Profissionais			
		41 (100,0)	13 (100,0)	2 (100,0)	26 (100,0)
**Região**					
	Norte	-	-	-	-
	Nordeste	11 (26,8)	4 (30,8)	-	7 (26,9)
	Centro-Oeste	4 (9,7)	2 (15,4)	-	2 (7,7)
	Sul	6 (14,6)	2 (15,4)	-	4 (15,4)
	Sudeste	18 (43,9)	5 (38,5)	2 (100,0)	11 (42,3)
	Rede (mais de uma região)	2 (4,9)	-	-	2 (7,7)
**Ano de início**					
	1970–2009	6 (14,6)	4 (30,8)	-	2 (7,9)
	2010–2016	27 (65,8)	8 (61,5)	1 (50,0)	17 (65,3)
	2017–2021	8 (19,5)	1 (7,7)	1 (50,0)	7 (26,9)

Foi identificada a oferta de 1.059 disciplinas em algum ano do quadriênio 2017–2020. A ausência de disciplinas com conteúdo de epidemiologia foi observada em apenas três programas. Todos os programas acadêmicos apresentaram pelo menos uma disciplina conceitual da área, enquanto nos programas profissionais essa oferta foi menos frequente (61,0%). Disciplinas focadas em coleta, processamento e análise de dados também foram mais frequentes nos programas acadêmicos (92,6%) do que nos profissionais (68,3%). As disciplinas com conteúdo de vigilância foram menos frequentes, presentes em apenas 33,3% dos programas acadêmicos e 26,3% dos profissionais. Disciplinas voltadas aos serviços e sistemas de saúde estiveram presentes em mais da metade dos programas acadêmicos e profissionais (55,5 e 53,6%, respectivamente). Por fim, os programas acadêmicos destacaram-se na oferta de disciplinas temáticas (90,7%), em contraste com os programas profissionais, em que apenas 41,5% ofereceram esse tipo de disciplina (Tabela 2).

**Tabela 2:** Distribuição dos programas de pós-graduação acadêmicos e profissionais segundo a oferta de disciplinas de epidemiologia por eixo temático. Brasil, 2021.

	Total n (%)	Saúde coletiva/pública n (%)	Epidemiologia n (%)	Com foco (como saúde da criança, ambiente…) n (%)
	Programas acadêmicos			
	54 (100,0)	38 (100,0)	5 (100,0)	11 (100,0)
Conceituais da área	54 (100,0)	38 (100,0)	5 (100,0)	10 (90,9)
Relacionadas a coleta, processamento e análise de dados	50 (92,6)	36 (94,7)	4 (80,0)	10 (90,9)
Vigilância	18 (33,3)	14 (36,8)	1 (20,0)	3 (27,3)
Voltadas a serviços e sistemas de saúde	30 (55,5)	19 (50,0)	4 (80,0)	6 (54,5)
Temáticos	49 (90,7)	31 (81,6)	5 (100,0)	11 (100,0)
	Programas profissionais			
	41 (100,0)	13 (100,0)	3 (100,0)	25 (100,0)
Conceituais da área	25 (61,0)	11 (84,6)	2 (66,7)	12 (48,0)
Relacionadas a coleta, processamento e análise de dados	28 (68,3)	10 (76,9)	2 (66,7)	15 (60,0)
Vigilância	11 (26,8)	5 (38,5)	2 (66,7)	4 (16,0)
Voltadas a serviços e sistemas de saúde	22 (53,6)	12 (92,3)	1 (33,3)	11 (44,0)
Temáticos	17 (41,5)	6 (46,1)	3 (100,0)	8 (32,0)

A maioria dos docentes e discentes concordou que os conteúdos básicos e obrigatórios eram suficientes para a adequada formação em epidemiologia. Foram observadas diferenças expressivas em relação aos conteúdos de pesquisa epidemiológica e aprofundamento temático: enquanto 73% dos discentes consideraram adequados os conteúdos de pesquisa, apenas 41% dos docentes concordaram com a assertiva. Por sua vez, 69% dos discentes avaliaram positivamente as possibilidades de aprofundamento na área, e apenas 46% dos docentes concordaram com essa afirmativa. Além disso, cerca de 40% dos docentes consideraram que o conteúdo de fundamentos da pesquisa epidemiológica e de vigilância epidemiológica não era suficiente para a adequada formação em epidemiologia (Figura 2).

**Figura 2:** Distribuição dos relatos dos docentes e discentes quanto aos conteúdos ministrados nas disciplinas de epidemiologia nos cursos de pós-graduação em Saúde Coletiva. Brasil, 2021.

O uso frequente ou muito frequente de metodologias ativas no ensino foi relatado por 58% dos docentes e 45% dos discentes. As atividades de análise de dados e o uso de *softwares* livres no ensino foram amplamente referidos por ambas as categorias (76 e 72% dos docentes; e 85 e 76% dos discentes, respectivamente). Atividades vinculadas a problemas reais da SC e a políticas públicas foram apontadas como realizadas com frequência ou muita frequência por mais de 80% dos docentes e cerca de 65% dos discentes. As atividades diretamente relacionadas à gestão ou aos serviços de saúde foram mencionadas como frequentes ou muito frequentes por 57% dos docentes e 56% dos discentes (Figura 3), indicando uma participação semelhante entre os dois grupos.

**Figura 3:** Distribuição de metodologia de ensino e uso de *software* por docentes atuantes em disciplinas de epidemiologia na pós-graduação e graduação em Saúde Coletiva. Brasil, 2024.

Ao serem questionados sobre as barreiras que comprometem o ensino de epidemiologia na pós-graduação, os participantes do estudo citaram como as mais frequentes as deficiências de conhecimento prévio dos discentes, especialmente em matemática, escrita científica e formação básica em epidemiologia e bioestatística. A heterogeneidade na formação prévia dificulta a compreensão de conceitos fundamentais e o domínio de análises mais complexas. Também foram relatadas limitações institucionais, como escassez de recursos didáticos e carga horária insuficiente para abordagem dos conteúdos essenciais pelos docentes.

Os principais facilitadores do aprendizado em epidemiologia mencionados pelos docentes se referiram ao acesso à infraestrutura e a recursos institucionais. Destacam-se o uso de *softwares* livres e pacotes estatísticos, a boa infraestrutura física (laboratórios de informática, salas de aula, bibliotecas), além da disponibilidade de dados locais e tecnologias educacionais. Também foram citadas como estratégias facilitadoras a adoção de métodos de ensino ativo, atividades práticas e a flexibilidade no formato das aulas, incluindo modalidades presencial, remoto ou híbrida (dados não apresentados em tabelas ou quadros).

Do ponto de vista dos pós-graduandos, as principais barreiras ao aprendizado nas disciplinas de epidemiologia dizem respeito à didática tradicional adotada por parte dos docentes, à predominância de métodos expositivos e ao uso de estratégias avaliativas pouco alinhadas com o processo formativo. Também foram mencionadas dificuldades relacionadas à limitação da carga horária para aprofundamento dos conteúdos e a resistência de alguns docentes de incorporar novas tecnologias e metodologias de ensino. Em contrapartida, os principais facilitadores do aprendizado se relacionaram à formação e à qualidade didática dos professores, à adoção de metodologias ativas e participativas e ao uso de exemplos práticos vinculados a contextos reais de saúde pública. Tais elementos são valorizados por favorecerem maior engajamento discente, melhor compreensão dos conteúdos e conexão entre teoria e prática, o que reforça a relevância de práticas pedagógicas mais dinâmicas, contextualizadas e centradas no estudante (dados não apresentados em tabelas ou quadros).

## DISCUSSÃO

O presente estudo mostrou que ainda há maior concentração dos programas de pós-graduação em SC nas regiões Sudeste e Nordeste, com maior diversidade temática no sudeste. A análise curricular revelou que, embora a maioria dos programas ofereça disciplinas conceituais e metodológicas, há menor oferta de conteúdos voltados à vigilância em saúde, especialmente nos programas profissionais. Além disso, foram destacados desafios na formação por docentes e discentes relacionados à heterogeneidade da formação prévia, à carga horária insuficiente e à didática tradicional, ao mesmo tempo que foram apontados fatores facilitadores, como o uso de *softwares* livres e a incorporação de atividades voltadas para problemas relacionados diretamente com políticas ou programas de saúde.

Também foi observada maior oferta de disciplinas de epidemiologia nos PPG acadêmicos, quando comparados aos PPG profissionais, mostrando sua importância enquanto eixo estruturante da área. Historicamente, o ensino de epidemiologia no Brasil é voltado para a saúde pública^4,7^. Atualmente, a epidemiologia é fundamental para a vigilância em saúde, coordenação de respostas a emergências em saúde pública e outras prioridades regionais e globais^4,8,9^.

Ressalta-se que menos da metade dos programas acadêmicos e profissionais oferece disciplinas voltadas à vigilância, e cerca de metade deles contempla disciplinas relacionadas aos serviços e sistemas de saúde, indicando uma formação técnica e política ainda limitada nesse eixo. O V Plano Diretor para o Desenvolvimento da Epidemiologia no Brasil (2025–2029) propõe resgatar a centralidade da vigilância no ensino da epidemiologia e fortalecer sua articulação com os serviços de saúde e com práticas intersetoriais.

Segundo Lau et al.^10^, a formação em epidemiologia deve contemplar, além de métodos analíticos, a aplicação prática em cenários reais, a comunicação dos resultados e a integração com os serviços. Os autores propuseram uma estrutura básica para formação em epidemiologia visando manter esse campo do conhecimento como fundamental para o enfrentamento de novos desafios da saúde pública. Considerou-se na proposta que o eixo de epidemiologia aplicada deve incluir não apenas desenhos de estudo e análises estatísticas sofisticadas, mas também contemplar aspectos da vigilância em saúde, coleta de dados primários, manipulação e análise de dados coletados na rotina dos serviços, além de uma robusta formação em epidemiologia descritiva e de campo.

Além disso, cerca de 40% dos docentes consideraram que o conteúdo voltado aos fundamentos da pesquisa epidemiológica e da vigilância não é suficiente para garantir a formação adequada na área, o que revela uma percepção crítica importante sobre lacunas estruturais nos currículos dos programas. Esse dado dialoga diretamente com as reflexões de Aquino^11^, que parecem permanecer atuais. A autora apontou que, apesar da expansão da pós-graduação e da intensificação da produção científica, ainda persiste um modelo formativo excessivamente centrado em disciplinas teóricas e desvinculado da prática investigativa concreta. Para superar esse quadro, Aquino^11^ defende a adoção de estratégias pedagógicas mais integradas à pesquisa que envolvam os alunos em experiências coletivas, como consórcios de pesquisa, e favoreçam a formação em contextos aplicados, socialmente relevantes e conectados às necessidades do SUS. Os resultados aqui apresentados sugerem que tais estratégias ainda não são amplamente incorporadas, especialmente nos programas profissionais, e apontam para a necessidade de um reposicionamento institucional mais consistente e direcionado a uma formação crítica, aplicada e comprometida com a realidade sanitária do país.

Conforme destacado no V Plano Diretor para o Desenvolvimento da Epidemiologia no Brasil (2025–2029), é necessário superar currículos fragmentados e desarticulados das necessidades do SUS na formação em SC. Essa crítica é reforçada por Barata^3^, ao apontar que o ensino de pós-graduação na área ainda carece de maior articulação com as demandas do sistema de saúde. Em consonância, Werler et al.^12^, ao discutir o ensino da epidemiologia em âmbito internacional, defendem a construção de currículos mais integrados, críticos e voltados à aplicação do conhecimento em contextos reais, perante os novos desafios científicos e sociais. No presente estudo, apesar da ampla presença de disciplinas conceituais e temáticas, especialmente nos programas acadêmicos, isso não garante, por si só, uma formação crítica e integrada às necessidades da população e dos sistemas de saúde. Assim, a formação crítica e técnica de epidemiologistas deve ser orientada não apenas pela excelência metodológica, mas também pela capacidade de atuação e adaptação em cenários complexos e de transformação da realidade social.

A crescente incidência de desastres naturais e emergências sanitárias, por exemplo, tem ampliado o escopo de atuação da epidemiologia, exigindo profissionais capacitados para lidar com cenários de alta complexidade e risco coletivo. A revisão de Liu et al.^13^ evidencia que a epidemiologia de desastres vem se consolidando como um campo estratégico, com aumento significativo na produção científica e destaque para temas como vigilância pós-desastre, avaliação de riscos à saúde, resposta rápida e uso de ferramentas analíticas para subsidiar a tomada de decisão. Esses avanços reforçam a necessidade de incorporar competências específicas na formação em epidemiologia.

O ensino da epidemiologia no Brasil tem passado por transformações significativas, especialmente no que se refere à adoção de metodologias ativas que buscam tornar o processo de aprendizagem mais dinâmico, participativo e centrado no estudante. Os resultados mostraram que as atividades práticas com base territorial têm sido incorporadas; cerca de 80% dos docentes disseram trazer para a sala de aula atividades voltadas para problemas reais da SC relacionados diretamente com políticas ou programas de saúde. Como defendido no V Plano Diretor para o Desenvolvimento da Epidemiologia no Brasil (2025–2029), essas atividades podem aproximar o ensino das demandas reais do SUS e populações vulnerabilizadas.

Lima et al.^14^, ao relatarem a experiência no ensino da epidemiologia, apontam para o uso de metodologias ativas, como aprendizagem baseada em problemas, sala de aula invertida e mapas mentais em disciplinas de graduação. Como resultados, os autores evidenciaram ganhos importantes no engajamento discente, na fixação dos conteúdos e no fortalecimento do vínculo entre professores e alunos, no entanto também ressaltam limites estruturais, como a carga horária restrita e a rigidez dos conteúdos teóricos, que dificultam a aplicação plena dessas metodologias. Esses achados reforçam a necessidade de se repensar os currículos, promovendo maior flexibilidade pedagógica e valorização de abordagens críticas e participativas.

A concentração dos programas de pós-graduação nas regiões Sudeste e Nordeste evidencia a disparidade no território brasileiro, destacando que a Região Sudeste ainda apresenta a maior diversidade temática no ensino de epidemiologia, reforçando desigualdades regionais quanto à oportunidade de formação especializada, uma vez que a concentração dos cursos de graduação em SC está em outras regiões. A maioria das graduações em SC entre 2008 e 2014 se concentrava na Região Nordeste e atualmente se concentra na Região Norte, por causa da abertura de cursos e universidades, com destaque para as públicas, que abrangem 68,5% dessas graduações^15^.

Por fim, destaca-se a necessidade de se desenvolver, de forma mais robusta, o eixo de comunicação científica no âmbito de divulgação de informações corretas e seguras para a população. Essa demanda foi evidenciada durante a pandemia de COVID-19^4^, mas é reforçada em menor proporção em relação à pandemia, por temas relativos a vacinação, mudanças climáticas e doenças emergentes e reemergentes. Essa estratégia contribuirá para formar epidemiologistas capazes de dialogar com diferentes públicos e combater a desinformação, incluindo em temas complexos como vacinação, mudanças climáticas e doenças emergentes.

Este estudo concentrou-se exclusivamente nos PPG *stricto sensu* da área de SC da Capes, com o objetivo de analisar em profundidade a inserção da epidemiologia nesse campo específico de formação. Reconhece-se, no entanto, que a epidemiologia também ocupa papel central em cursos de graduação da área da saúde e em PPG de outras áreas de avaliação, como medicina I e II, enfermagem, nutrição, entre outras, além de cursos em outras modalidades de formação. A delimitação adotada foi uma abordagem metodológica necessária para assegurar maior rigor e profundidade na análise, embora não esgote o debate sobre os desafios e as potencialidades do ensino de epidemiologia no contexto mais amplo da formação em saúde. Em contrapartida, trata-se da primeira iniciativa de traçar o perfil dos cursos e a visão dos docentes e discentes dos PPG em relação ao ensino da epidemiologia.

Os achados deste estudo indicam avanços importantes, como a presença de disciplinas estruturantes, adoção de estratégias de ensino com atividades relacionadas diretamente com políticas ou programas de saúde e a incorporação de metodologias ativas, além de evidenciar lacunas significativas na formação crítica e aplicada em epidemiologia, sobretudo nos programas profissionais.

Diante desse cenário, fazem-se necessários o fortalecimento do eixo de vigilância e pesquisa aplicada nos currículos e a valorização de estratégias pedagógicas integradas aos serviços de saúde. Recomendam-se, ainda, maior investimento em infraestrutura didática, apoio docente e articulação interprogramas, de modo a estimular experiências colaborativas, como ensino em rede. Por fim, ressalta-se a importância de incorporar conteúdos relacionados à comunicação científica, à análise de dados em contextos reais e ao enfrentamento das desigualdades regionais, como dimensões essenciais para a formação de epidemiologistas capazes de responder aos desafios atuais da saúde no Brasil.

## Data Availability

O conjunto de dados que dá suporte aos resultados deste estudo não está disponível publicamente.

## Material Suplementar

Material Suplementar 1
